# Assessing three altruism facets by economic games and self-report: a multitrait-multimethod investigation

**DOI:** 10.1038/s41598-026-46603-w

**Published:** 2026-04-03

**Authors:** Lucie Binder, Martin Schultze, Frances S. Chen, Sabine Windmann

**Affiliations:** 1 Department of Psychology, Goethe University, Theodor-W.-Adorno Platz 6, 60323 Frankfurt, Germany; 2 Department of Psychology, University of British Columbia, Vancouver, British Columbia Canada

**Keywords:** Altruism, Economic games, Self-report measures, Validity, Social preferences, Evolution, Psychology, Psychology

## Abstract

Reliable and valid measurement of the various components of prosociality calls for tools that capture its diverse behavioral expressions. Here, we evaluate how economic game measures derived from the Dictator Game, Public Goods Game, and Ultimatum Game as well as a novel Third-Party Intervention Paradigm correspond with their self-reported counterparts within a design informed by a multitrait–multimethod approach. The self-report scales described Help Giving, Peer Punishment, and Moral Courage as behavioral traits in real life. Each game decision was regressed on all three of these scales using data from 22 studies. Convergent validity emerged, with the strongest associations for help giving and the weakest for peer punishment. Discriminant validity was evidenced by the lack of significant cross-correlations, with one minor exception regarding the Peer Punishment scale and the Dictator Game. Overall, the findings support the distinctiveness of the facets, particularly for help giving and moral courage, while highlighting challenges in capturing punishment-related altruism. While several procedures for improving correspondence are discussed, the descriptively higher correlations within methods than across traits suggest a persistent gap in criterion validation.

## Introduction

Today’s societies are confronted with large-scale challenges such as climate change, pandemics, and rising authoritarianism fueled by online spread of misinformation^1–5^. These crises are rooted in the conflict between short-term self-interest and long-term collective welfare^6–9^. Addressing these dilemmas with scientific methods requires translating real-world cooperation problems into reliable and valid research paradigms that allow researchers to reconstruct the relevant system characteristics from individual decisions^8,10–12^. Developing and disseminating valid and efficient tools for measuring prosociality in all its facets is an essential prerequisite for this endeavor. By enabling the testing of theoretical assumptions, such research paradigms provide the foundation for designing effective interventions and policies^6,8,13–16^.

From a psychological perspective, prosocial behavior, and altruism in particular, has been broadly defined as actions *intended* to benefit others^17–21^. Research in this tradition has emphasized the role of personality traits such as extraversion^22^, openness^22^, agreeableness^23^, honesty-humility^24,25^, empathy^17,26–29^, narcissism^30–34^, emotional intelligence^35,36^, moral reasoning^37–39^, and risk-taking^40,41^. Developmental factors have also been shown to shape prosocial tendencies across the lifespan^37–39^. Thus, psychology largely describes individual differences in prosociality as trait-level dispositions assessed via self-report^42^.

Self-report measures used to assess prosociality include the Prosocialness Scale^43^, the Prosocial Tendencies Measure^44^, the Interpersonal Reactivity Index^27^, and inverse scales for antisocial traits like Machiavellianism^45,46^. These questionnaires mostly assess inner states and motives for helping or sharing with others. To our knowledge, only two instruments exclusively assess prosocial *behaviors*, specifically, altruistic behaviors. These are the Self-Report Altruism (SRA) Scale^47^, which measures past helping and donating behavior, and the Facets of Altruistic Behaviors (FAB) scale^48^ that additionally assesses confrontational facets, namely moral courage (more recently termed countercontrol, see Windmann^49^) and norm-enforcing peer punishment. Other attempts to capture confrontational forms of altruism with self-report scales have remained preliminary and untested^50,51^.

Self-report questionnaires are practical tools for the assessment of real-life behaviors and attitudes that allow for psychometric evaluation of core assumptions regarding the structure and relations of latent dimensions. As such, they can shed light on underlying mechanisms and enable reliable comparisons across individuals and contexts^47,48,52,53^. Nevertheless, they also have critical limitations. First, they rely heavily on language, which makes them difficult to use for cross-cultural comparisons or in individuals with language impairments. Second, they depend on subjective judgment^54,55^, and therefore require conscious awareness as well as realistic comparison standards. Third, they are vulnerable to social desirability bias^56,57^, a problem that is particularly relevant for the assessment of prosociality and altruism.

In contrast to the intention-focused assessment common in psychology, behavioral economics tends to rely on behavioral responses observed in paradigms derived from game theory as proxies for altruism, cooperation, and norm-enforcing punishment^58–63^. Paradigms such as the Dictator Game, Ultimatum Game, and Prisoner’s Dilemma Game present participants with monetary incentives when trading off benefits for self against those of others, often played as one-shot interactions to avoid interactive dynamics. The assumption is that the choices made under such controlled conditions directly reveal underlying social preferences, without the need for additional measurement or interpretation.

Using such games, studies with large Western samples have shown that people who punish others are not the same as those who altruistically share with others^51,64–66^, see also^67^ for a Japanese sample], suggesting that altruistic punishment and altruistic rewarding are distinct social behavioral traits. In line with these interpretations, giving behavior in economic games has been found to correlate significantly with self-reported honesty-humility^68,69^, agreeableness^70–72^, openness^72^, and empathy^73,74^, while anger accompanies punishment^75–77^. Other studies have shown the influence of contextual factors on economic game decisions such as anonymity^78–80^, reputation formation^81^, social distance^82–86^, and social norms^87–91^. Game behaviors have further been linked to independently obtained objective indices of socio-structural conditions at the cultural level, including market integration^60,92^, affiliation with a world religion^92^, exposure to the Western Church, kinship ties, and prevalence of cousin marriage^93^. Finally, the preferences shown in the games relate to monitoring, sanctioning, and leadership-related behaviors in field settings^94–96^, as well as occupational choice into health care and public services^97–99^.

Taken together, these findings demonstrate that economic games can effectively capture both situational influences as well as individual, cultural, and societal factors influencing decision-making in social dilemmas in large-scale research studies. Their use as diagnostic tools for individual assessments, however, seems limited^51,65,70,100^. Relatively low retest reliabilities of *r*=.57–.63 over a six-week interval^70^ and *r*=.36 over a 124-day interval^51^ have been reported, values that fall short of psychological standards for diagnostic tests^101^. Since reliability is a prerequisite for validity, these values constrain the accuracy of the games for individual-level diagnostic purposes.

A related concern is that the empirical link between self-reported and behavioral measures of prosociality appears weak and inconsistent. This may partly reflect a genuine intention–behavior gap^102–104^, but it could also stem from conceptual disparities across research disciplines that may lead to different operationalizations and analyses. First, economists usually refer to ultimate definitions of behavior, while psychologists tend to adopt an intentionalist perspective^20,21,49^. Second, some formalized definitions of decomposed games, based on monetary payoff distributions, clearly differentiate altruistic from prosocial decisions and cooperation^105–107^, whereas verbal accounts tend to use these terms interchangeably. Third, latent factors such as personal moral values are central constructs in psychology, but have only very recently been allowed to enter economic equations^108^. Finally, even within the realm of empirical findings, inconsistencies prevail, because some studies have relied on insufficiently validated scales^50,51,109^, used theory-free items or item combinations^109,110^, and/or assessed motives and attitudes rather than concrete behaviors^43,44,111^. It thus remains unclear to what extent self-reports capture the same forms of altruistic behaviors that are examined by economic games and vice versa.

The present study compares behaviors observed in three types of well-established economic games with psychometrically evaluated self-report short scales that were specifically designed to represent those same behaviors in real-life contexts^48^. For the games (see Table 1), the Dictator Game was used to capture altruistic sharing, measured by the amount of money that one player unilaterally allocates to another^79,109,112,113^. A Public Goods Game setting with a punishment option was most often used (in 1,548 of the 2,224 punishment-related decisions) to assess costly norm-enforcing by punishing those who fail to contribute their fair share^51,114,115^. The Ultimatum Game, a simpler alternative, was used less often (in 676 of the 2,224 punishment-related decisions) to capture costly norm-enforcing by rejecting unfair offers^116,117^. Because no game exists for moral courage behavior, a novel Third-Party Intervention Paradigm, as introduced by Windmann et al.^48^, was used to assess morally courageous behavior in the form of confronting an authority figure acting unfairly (see Methods for details).

To determine the link between the self-report and behavioral assessment of the three altruism constructs, we analyzed the convergent and discriminant validity of the Facets of Altruistic Behaviors (FAB) subscales^48^ and the aforementioned economic games. Specifically, we regressed each game behavior on the subscales of the FAB scale. Notably, that scale was developed using an algorithmic approach that enabled optimization of psychometric properties^118^. Unlike other self-reports that measure attitudes, motives, and feeling states^27,43,44^, the items of the FAB scale provide descriptions of altruistic *behaviors* along three dimensions: First, the Help Giving subscale (HG) assesses the habitual sharing of resources (e.g., money, time, information) with needy or deserving others. Second, the Peer Punishment subscale (PP) captures the coordination of anonymous punishment of unfair group members, as done in the Public Goods Game paradigm. Third, the Moral Courage subscale (MC) measures the likelihood of proactively confronting powerful others who violate one’s moral convictions.

Estimating the convergent and discriminant validity of these three altruism facets measured by the FAB scales on the one hand and economic game behaviors on the other essentially follows the logic of a multitrait-multimethod approach^119^. The design enabled us to examine whether HG reliably predicts the amount shared in the Dictator Game; PP reliably predicts punishment in the Public Goods Game as well as rejection likelihood and the Minimal Acceptable Offer (MAO) in the Ultimatum Game; and MC reliably predicts the likelihood of confrontational responding in the Third-Party Intervention Paradigm. At the same time, we were able to examine any undesired cross-correlations between games and scales to determine how facet-specific each of the predictions are.

Data from 5,806 participants across 22 studies who completed the FAB scale were analyzed, allowing us to estimate factor scores and improve measurement precision. A subset of these participants (*n* = 1,843) additionally played the economic games, enabling us to link behavioral and self-report measures. We used a (generalized) linear model approach with mixed effects (where applicable) to account for the nested structure of the data. We thereby predicted each game behavior from the pertinent FAB subscale while retaining the other two subscales in the model to be able to evaluate subscale-specificity in predicting the criterion behavior.

Moreover, we evaluated the overall pattern of pairwise correlations of all self-report subscales and game behaviors. We expected strong positive correlations (*r* >.30^120^ within facets across methods, which would reflect convergent validity. By contrast, we expected comparably weak correlations (*r* <.20) between non-corresponding facets within and across methods, indicating discriminant validity. The games were played anonymously and one-shot, apart from hypothetical practice trials in the Public Goods Game. Accordingly, HG and PP items were also phrased anonymously; for MC this was not always possible (see Supplement S1 for English translations).

Finally, we investigated the role of another factor that psychology and economics treat differently: the role of deception. In economics, game theoretical decisions are almost always fully incentivized, and deception is essentially seen as malpractice^121,122^. By contrast, psychologists more often weigh costs against benefits when deciding about deception^123,124^. In fact, at least half of the 25 most influential experiments in Psychology listed by Fescoe^125^ involve deception or grossly incomplete participant information about the study’s true purpose.

In our studies, participants were either informed that the other players in the economic games were fictitious, or they were initially deceived about their authenticity and later debriefed. This variation was independent of monetary incentives. To determine pragmatically whether deception changes participants’ behaviors and whether it moderates the association between self-report and game behavior, we included a binary variable in our models corresponding to whether or not the study entailed deception.

All data, analysis scripts, preregistrations, and supplementary files are available at: https://doi.org/10.17605/OSF.IO/T5MF2  

**Table 1:** Overview of experimental measures.

Facet	Paradigm	Measures
Helping	Dictator Game	Likelihood to share and amount of sharing.
Punishment	Public Goods Game	Likelihood to punish and amount invested into punishment.
	Ultimatum Game	Likelihood to reject an unfair offer and Minimal Acceptable Offer.
MC behavior	Third-Party Intervention	Likelihood to intervene by sending a message.

Note. MC stands for moral courage.

## Results

The measurement model of the three-factorial FAB scale met the cutoff criteria for both RMSEA and SRMR, with SRMR = 0.04 and RMSEA = 0.05. The CFI was slightly lower than the cutoff with CFI = 0.94. Latent correlations between factors were *r* =.33 for HG and PP, *r* =.60 for HG and MC, and *r* =.63 for PP and MC.

### Prediction of game behavior

The parameters for all models are presented in Table 2. The ICCs for the intercept-only models were ICC = 0.156 and ICC = 0.026 for the logit and linear model of Dictator Game sharing, respectively. The logit model showed that a one-unit increase in HG (which is here a one standard deviation increase) was associated with an almost fivefold increase in the odds of sharing money in the Dictator Game, with an odds ratio of 4.99, while controlling for the other predictors. In the linear mixed model that included only those participants who did indeed share money in the Dictator Game, HG also predicted sharing behavior in the Dictator Game significantly. A one standard deviation increase in HG was associated with a 40.1% higher shared amount on the original scale. Conversely, a one standard deviation increase in PP was significantly associated with an 8.7% lower shared amount.

For punishment in the Public Goods Game, the ICCs for the intercept-only models were ICC = 0.053 and ICC = 0.023 for the logit and linear model, respectively. We did not find any significant effects of PP or any other predictor on the likelihood to punish or the costs invested in punishment. In the Ultimatum Game, an increase of one standard deviation in PP was associated with an increase of 0.60 points in the Minimal Acceptable Offer (MAO), while holding all other variables constant. Conversely, the MAO decreased by 0.39 points when deception occurred compared to when it did not. However, PP did not significantly predict the likelihood to reject.

For Third-Party Intervention, the ICC for the intercept-only model was ICC = 0.057. A one standard deviation increase in MC was associated with 2.18-times higher odds of intervening, while holding all other variables constant.

### Correlations within and across methods

**Fig. 1:**
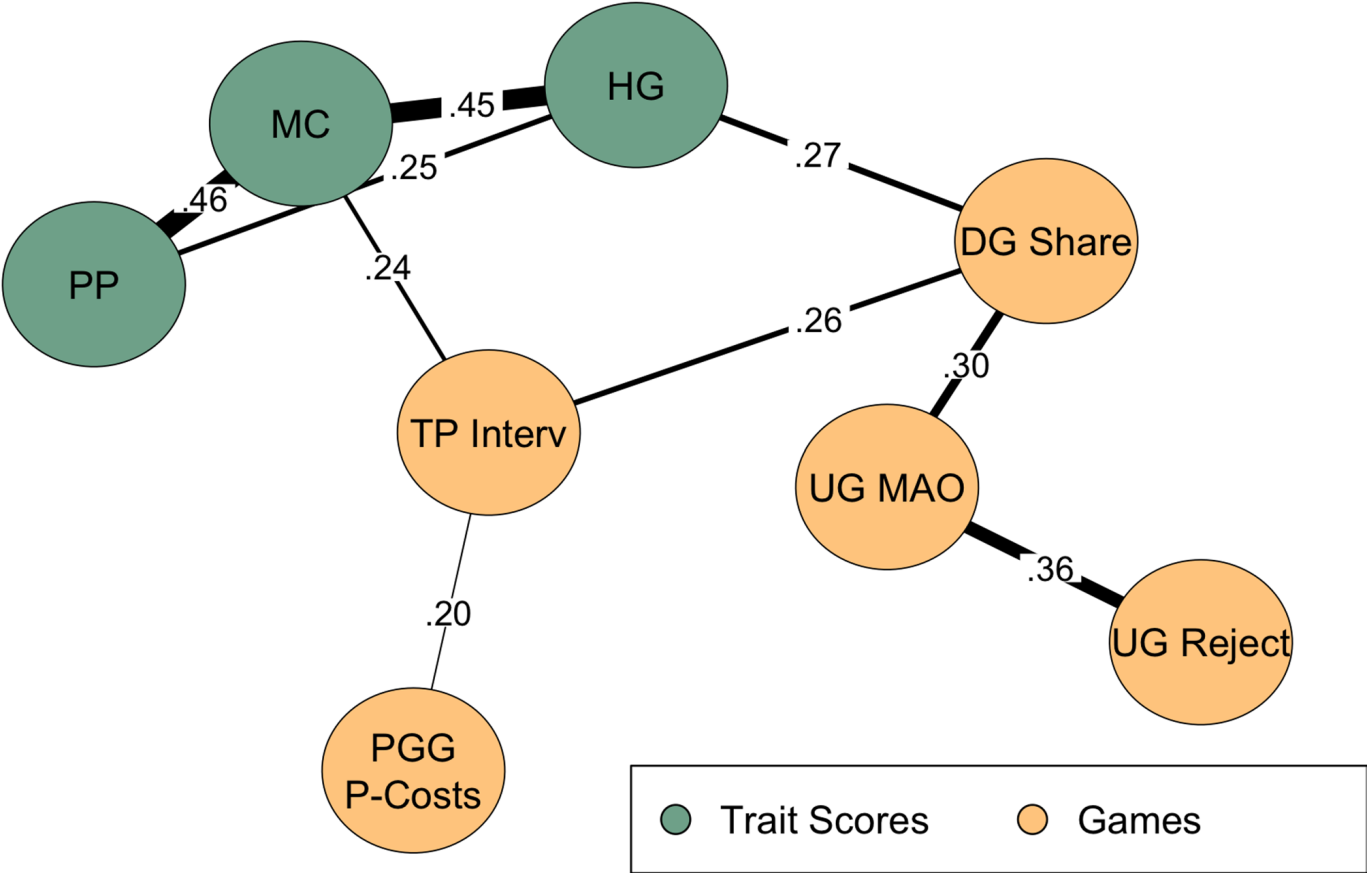
Pairwise correlations of self-report subscale scores and game behaviors. *Note*. Only correlation coefficients higher than *r* =.20 are shown^120^. For the games, decisions (binary) and amounts (continuous) were combined where applicable, unlike in the analyses shown in Table 2. Spearman coefficients are shown for reasons of comparability^51,70,100,110^ and because a more suitable alternative is lacking, but note that these may be biased given the extremely skewed distributions typical of Dictator Game sharing and punishment costs, both of which include a large number of zero values. All reported correlations are significant at *p* <.001. HG = Help Giving subscale score, PP = Peer Punishment subscale score, MC = Moral Courage subscale score, DG Share = Amount shared in the Dictator Game (including zeros), PGG P-Costs = Punishment costs in the Public Goods Game (including zeros), UG Reject = Likelihood to reject in the Ultimatum Game, UG MAO = Minimal Acceptable Offer in the Ultimatum Game, TP Interv = Likelihood to intervene in the Third-Party Intervention. The full correlation matrix is presented in Supplement S2 in the online supplement.

An overview of the Spearman correlations within and across methods are presented in Fig. 1, with only medium-sized coefficients of *r* >.20 indicated. Highest correlations are observed between FAB subscale scores. Regarding convergent associations across methods, HG and Dictator Game sharing were significantly correlated, *r* =.27, *p* <.001, as well as MC and third-party intervening, *r* =.24, *p* <.001. PP did not show correlations higher than .20 with the corresponding game behaviors. The complete correlation matrix can be found in Supplement S2 in the online supplement.

**Table 2:** Model parameters for all behavioral outcomes that measure helping, punishment, and morally courageous behavior.

	Helping				Punishment							MC Behavior	
	Likelihood to Share (Logit Model)		Shared Amount (Linear Model)		Likelihood to Punish (Logit Model)		Costs Invested in Punishment (Linear Model)		Likelihood to Reject (Logit Model)		MAO (Linear Model)	Likelihood to Intervene (Logit Model)	
	*Estimate* (*SE*)	*OR* [95% CI]	*Estimate* (*SE*)	D% [95% CI]	*Estimate* (*SE*)	*OR* [95% CI]	*Estimate* (*SE*)	D% [95% CI]	*Estimate* (*SE*)	*OR * [95% CI]	*Estimate* (*SE*)	*Estimate* (*SE*)	*OR * [95% CI]
(Intercept)	1.24** (0.23)	3.44 [2.19, 5.42]	1.21** (0.06)	236.18 [197.54, 279.85]	–0.46* (0.22)	0.63 [0.41, 0.97]	0.79** (0.05)	119.63 [99.84, 141.40]	–0.83* (0.14)	0.44 [0.33, 0.57]	4.08** (0.09)	–0.61** (0.19)	0.54 [0.37, 0.79]
Factor scores													
Help Giving	1.61** (0.23)	4.99 [3.16, 7.88]	0.34** (0.09)	40.05 [18.27, 65.85]	0.06 (0.20)	1.06 [0.72, 1.56]	–0.01 (0.09)	–1.37 [–17.13, 17.38]	–0.72 (0.36)	0.49 [0.24, 0.99]	0.01 (0.30)	0.32 (0.22)	1.38 [0.90, 2.11]
Peer Punishm.	–0.17 (0.12)	0.85 [0.67, 1.07]	–0.09* (0.04)	–8.67 [–15.96, –0.74]	–0.01 (0.11)	0.99 [0.80, 1.22]	0.08 (0.05)	7.84 [–1.78, 18.41]	0.25 (0.23)	1.28 [0.82, 2.03]	0.60** (0.16)	–0.14 (0.11)	0.87 [0.69, 1.08]
Moral Courage	–0.19 (0.18)	0.82 [0.58, 1.17]	0.06 (0.07)	5.90 [–6.98, 20.56]	0.21 (0.16)	1.23 [0.90, 1.69]	–0.00 (0.07)	–0.05 [–13.23, 15.14]	0.06 (0.31)	1.06 [0.58, 1.93]	0.01 (0.23)	0.78** (0.18)	2.18 [1.53, 3.12]
Game design													
Deception	–0.46 (0.42)	0.63 [0.28, 1.42]	0.14 (0.12)	14.85 [–8.62, 44.34]	0.17 (0.40)	1.18 [0.55, 2.57]	–0.09 (0.09)	–8.36 [–23.66, 10.01]	–0.00 (0.21)	1.00 [0.66, 1.51]	–0.39* (0.18)	0.25 (0.38)	1.29 [0.62, 2.69]
Factor Score: Deception	0.15 (0.43)	1.16 [0.50, 2.71]	–0.15 (0.18)	–14.19 [–40.19, 23.10]	0.25 (0.21)	1.28 [0.85, 1.94]	–0.03 (0.10)	–2.85 [–20.42, 18.60]	–0.04 (0.28)	0.96 [0.56, 1.67]	–0.52 (0.30)	0.05 (0.29)	1.05 [0.60, 1.85]
Intercept (Study ID)	0.18 (0.43)		0.01 (0.11)		0.17 (0.41)		0.01 (0.07)		NA		NA	0.12 (0.35)	
Level-1 residuals	NA		0.48 (0.69)		NA		0.34 (0.58)		NA		NA	NA	
N of studies	6		6		6		6		3		4	6	
Observations	1,574		1,099		1,548		664		450		676	1,536	
Model Accuracy	0.71		NA		0.56		NA		0.68		NA	0.66	
Marginal R^2^ / Conditional R^2^	0.087 / 0.135		0.034 / 0.059		0.010 / 0.058		0.011 / 0.026		0.015		0.050	0.079 / 0.112	

Note. Marginal and conditional R^2^ were estimated following Nakagawa et al.^126^ For the logit model on likelihood to reject, we report Tjur’s^127^ R^2^, for the linear regression model on MAO, we report the proportion of variance explained. Significant estimates are indicated in bold font. In the interaction of factor score x deception, the factor score always refers to the subscale that was aimed to assess the same facet as the game behavior. OR = Odds ratio, △% = percent change on the original scale. *p < .05; **p < .01.

## Discussion

The study examined the convergent and discriminant validity of three facets of altruism, measured via self-report and economic game decisions, following the logic of a multitrait-multimethod approach^119^. Behavioral measures were taken from the Dictator Game, two punishment games (Public Goods Game and Ultimatum Game), and the Third-Party Intervention Paradigm. The self-report measures captured the traits Help Giving (HG), Peer Punishment (PP), and Moral Courage (MC) which are described as behaviors rather than attitudes or feelings. Assuming that the three facets represent distinct constructs, we expected to observe significant convergent associations within constructs across methods, but low discriminant associations between constructs within methods, and especially between constructs across methods. Data pooled across 22 studies were considered in linear mixed model analyses including random effects for study (except for Ultimatum Game behaviors, see Methods), a fixed effect for “deception” (real vs. forged interactions), as well as the interaction of deception with self-report measures to quantify how deception influenced the relationship between the two methods within each trait.

When game behaviors were regressed onto the factor scores of the self-reported facets to determine the degree of variance overlap (i.e., across methods), we observed significant positive coefficients. First, self-reported HG positively predicted Dictator Game decisions, both in terms of the likelihood of sharing and the amount shared. Second, self-reported MC positively predicted the likelihood to intervene in the Third-Party Intervention Paradigm. Third, self-reported PP significantly predicted the Minimal Acceptable Offer (MAO) in the Ultimatum Game, albeit not the likelihood to punish, the amount invested in the punishment in the Public Goods Game, or the likelihood to reject unfair offers in the Ultimatum Game.

By contrast, the other two factor scores, those that were not specific for the predicted game behavior, did not yield significant coefficients in any of the analyses, confirming our expectation of discriminant validity of the three altruism facets. The only exception was the self-reported PP negatively predicting the amount shared in the Dictator Game (β = –0.09, *SE* = 0.04). Notably, this analysis included only those individuals who had already decided to share, indicating that among the group of sharers, higher self-reported PP was associated with a slightly reduced shared amount.

Taken together, our results confirm the expected pattern of convergent and discriminant validity of the altruism facets, at least for HG and MC. Game behaviors were positively predicted by their corresponding self-reported behavioral traits (with the exception of PP), but not by the two other traits, respectively. HG showed a clear, albeit modest, prediction of Dictator Game decisions: A one standard deviation increase on the HG score was associated with an almost fivefold higher likelihood of sharing in the Dictator Game, and a 40% higher amount given by those who did share something. This prediction reflects a Spearman correlation of *r* =.27 between the shared amount and HG score. While this correlation does not quite reach the “strong” threshold suggested by Funder and Ozer^120^ (*r* >.30), it exceeds the associations reported by Galizzi and Navarro-Martinez^128^ with regard to the Self-Report Altruism Scale^47^and those reported by Böckler et al.^100^ with regard to the Interpersonal Reactivity Index^111^ and the Prosocialness Scale^43^. However, our correlation is slightly below the coefficients reported by Kosfeld et al.^110^ (R^2^ = .096 ≙ *r* =.31 according to Table3) for single self-report items predicting their measure of Dictator Game giving, namely to a charity. Procedural, statistical, and sample characteristics may account for differences between the two result patterns. Of note, however, is that in the Kosfeld et al. study^110^, the `hypothetical Dictator Game´, a self-report item that essentially describes the same scenario as the game itself verbatim, was the strongest predictor of Dictator Game giving. Interestingly, the predictive power of that item was further increased when combined with other, qualitative self-report items describing altruistic choices. Such aggregation across multiple items is customary for psychological self-report measures like the FAB scale.

One caveat regarding a stand-alone hypothetical item is that it may inflate the correlation with actual game behavior by inducing a consistency bias, even though its association with real-life criteria may remain low^110,128,129^. To some extent, such a bias exists in all research linking self-report measures to criterion behavior. In the present study, we have attempted to minimize this bias by describing complex real-life scenarios in the FAB items and by investigating multiple traits at once. Additionally, we have separated self-report and behavioral assessments by approximately two weeks in most of our studies.

With regard to MC, prediction of the Third-Party Intervention Paradigm, which was specifically designed to measure moral courage as conceptualized in the FAB scale, was significant, with a pairwise correlation that can be considered modest, falling between “intermediate” and “strong” according to Funder and Ozer’s guidelines^120^. Notably, there is currently no other game for assessing moral courage, so that we are unable to compare our findings to other studies. Future studies could develop procedures that measure principled dissent in the face of social threat, such as risk of shame, ridicule, or ostracism. Another idea for assessment in the laboratory would be to “bribe” or “scare” the decision-maker away from a value-driven behavior or claim. Ecological validation criteria could include activism, whistleblowing, or other instances of resisting current practices for ethical reasons in professional or social contexts despite the risk of social conflict and degradation^49^.

Conceptually, moral courage differs from peer punishment in that it refers to the principled pursuit of prosocial values that may lie outside common norms, rather than to the concrete punishment of specific norm violations occurring among peers. Some self-report scales have indeed tried to capture a construct that resembles this definition, but these are not sufficiently evaluated^50^, are based at least partly on a different understanding of moral courage^130^, or refer to specific application fields^131,132^. In our view, a concise description of moral courage or principled dissent in behavioral terms is lacking both in the psychological and the economic literature^49^.

Concerning peer punishment, the observed association between game behaviors and the PP scale failed to meet our expectations, similar to what has been reported for negative reciprocity by Kosfeld et al.^110^. Not only did the PP score show relatively poor specificity in predicting punishment behavior due to its negative cross-correlation with the amount given in the Dictator Game (referring to amounts larger than zero); it also failed to show significant convergent correlation with (i) the likelihood to punish in the Public Goods Game, (ii) the amount invested in the punishment in the Public Goods Game, and (iii) the likelihood to reject an unfair offer in the Ultimatum Game. This is true despite the fact that PP factor scores were estimated on the basis of 5,806 data points, to increase the reliability of the estimate. Under these conditions, we would have expected relatively high power to detect significant predictions and raw correlations above *r* =.20^120^. While PP significantly predicted the MAO in the Ultimatum Game, a game measure that is usually interpreted in terms of peer punishment^51,110^, the pairwise correlation between the two was relatively low (*r* =.19).

In conjunction with results from other studies with the FAB scale^133,134^, items of the PP subscale may have to be revised to better mirror altruistic punishment under anonymous conditions in economic games. However, it is quite challenging to press into a single sentence the description of a Public Goods Game interaction starting from the observed violation of a group’s fairness norm and including the costly reaction of the actor under conditions of anonymity that results in the subtraction of the free rider’s resources. This problem is similar for rejection of unfair offers in the Ultimatum Game, especially when considered from a third-party perspective which is more important here than in the Public Goods Game because it helps to minimize self-concerned retaliation^135^. However, the PP items of the FAB scale were originally intended to reflect anonymous punishment of free riders in a collaborative group setting, as exemplified by the Public Goods Game.

During the construction of the PP subscale of the FAB scale, Windmann et al.^48^ presented the selection algorithm with 19 items describing acts of peer punishment in real life that entailed some variation while still capturing the essence of punishment of free riding. Based on model fit, including internal consistency, the algorithm selected a set of five items that now constitute the PP scale. All five items describe a group setting; two of them describe anonymous (“private” or “discrete”) acts of punishment, one the preparation of such acts (by monitoring deviant behavior), while the remaining two describe attempts to *coordinate *the punishment with other ingroup members. Given the adequately high internal consistency of the PP subscale, and its successful demarcation from the other two facets, we find it unlikely that the conceptual variation across the PP items might account for the relatively low correlations we found in the present study between the PP scale and punishment in the Public Goods Game. This interpretation is consistent with the findings of Falk et al.^109^ and Kosfeld et al.^110^ who also report relatively low convergent correlations of punishment survey items with punishment in economic games.

Another point that was noticed during item construction is that many descriptions of PP behavior tend to make the actor appear quite unlikable and unsympathetic. The behaviors come across as hostile and destructive, and most respondents would probably refrain from endorsing such socially unfavored, efficiency-reducing acts^135–139^. Precisely because of this potential to generate social tension and conflict, Guala^140^ suggested that peer punishment, in a strict sense, may not exist outside the imperative framework of experimental laboratory tasks. Ultimately, we think that the construct of peer punishment as it applies to real life may need to be reevaluated altogether to ensure its ecological plausibility^76,135,140–142^. Some promising attempts have been made with daily surveys^143^ and ecological momentary assessments^144^.

One generally important observation from the pattern of pairwise correlations (Fig. 1) is that the coefficients within methods tend to be higher than those within constructs. Self-reports, in particular, cluster together, as do the punishment games to some degree (compare refs^51,100^). This descriptive pattern is consistent with the findings of Böckler et al.^100^, who subjected various game decisions and questionnaire scores to factor analysis and identified one factor specifically for self-reports that was largely independent of the game behaviors. In that study, however, the questionnaire items did not describe behaviors as they do in the FAB scale, let alone confrontational ones, but rather feelings of care and empathy which primarily refer to helping. The present finding of a clustering among the three subscales of the FAB scale is a limitation from the perspective of criterion validation, i.e., within constructs and across methods.

Beyond these main results, the factor deception was included in the model analyses. We found that deception affected game behavior in only one measure, namely the MAO. When participants were led to believe that the other participants were real, the Minimal Acceptable Offer decreased, on average by 4%, compared to when participants knew that the other participants were fictitious. The finding corresponds with previous studies suggesting that people dislike punishing other real people^135,140,145^, which may have opportunistic or altruistic reasons in the Ultimatum Game. The finding may relate to other unexpected effects involving the MAO in the present study, including its significant positive correlation with Dictator Game giving. In both cases, participants provide a number that determines their own payment, which might feel more relevant to them than spending money on punishment of other players under anonymous conditions.

No other game behavior was significantly impacted by deception in our studies, while we acknowledge that the monetary stakes were relatively low overall (max. $1.25/€1.25 in all games, with participants receiving their share in all cases). Naturally, deception should always be kept at a minimum in scientific studies, and we caution against resorting to “deceiving about deception” tactics and other practices that may camouflage or understate the actual degree of deception^146–148^. The way deception is handled by the scientific community differs markedly between disciplines: In economics, on the one hand, it is essentially banned^121^, especially due to its long term impact on the participant pool^149^. Psychology, on the other hand, uses deception more broadly to avoid demand effects and other reactivity-related influences on participants’ behavior^123,124^.

Generally, cross-talk between economics and psychology could be improved, as suggested by illustrative evidence: In Batson’s^150^ article on the role of empathy in altruistic helping, only 6% of the cited references refer to economic journals, while 74% were published in psychology journals. Conversely, in the paper by Falk et al^.109^, 66% of the references are published in economic journals, while only 18% refer to psychology journals (see Supplement S3 for categorization). The limited cross-disciplinary communication may hinder researchers’ understanding of the relationship between self-reported and game-based behavioral measures.

In conclusion, how can we improve self-report measures of altruism to more accurately capture the behaviors of interest, whether they are shown online, in a laboratory setting, or in real life? We see three possibilities. The first one is constructing self-report scales with the explicit aim of maximizing convergent validity with economic games, if not real-life behaviors directly, and integrating this criterion into data-driven item generation and selection procedures^151^. Second, multiple trials should be aggregated to achieve higher psychometric quality compared to single-item measures. This holds for both self-reports^110^ and economic games^152^. Third, the theory behind the questionnaire construction needs to provide concise, operationalizable, and non-redundant definitions of the constructs of interest^153^, something that psychology sometimes struggles to achieve^154^.

In sum, our findings support the multidimensional view of altruistic behaviors and show that help giving, peer punishment, and moral courage are related yet distinct constructs. Although the latent factors of the FAB scale were moderately to strongly correlated, they differed in their ability to predict the different game behaviors, especially for help giving and moral courage. Our findings are thus consistent with earlier studies that have distinguished the three altruism facets based on their association with career choices^133^ and their sensitivity to variations of social distance^134^. Together, these results underscore the importance of distinguishing the facets of altruism both conceptually and empirically.

## Methods

### Participants

Across all studies, the overall sample consisted of 5,806 participants (age, *M* = 41.0 years, *SD* = 15.2, range: 18–94) with 3,141 female (54%) and 25 non-binary persons. Participants were either U.S. or German. In addition to responding to the Facets of Altruistic Behaviors (FAB) scale, a total of 1,843 participants (age, *M* = 43.9 years, *SD* = 15.2, range: 18–94; 934 female (51%) and 11 non-binary persons) in 6 of the 22 studies also took part in the economic games. To achieve representative samples, we used quotas for age, gender, and education in 11 studies, three of which were collected via MTurk and eight via panel providers. The remaining 11 studies investigated mainly students. Table 3 gives an overview on study characteristics. Importantly, the online supplement contains a detailed table of study characteristics at the study level as well as data, codebooks, preprocessing scripts, and preregistrations for each study.

**Table 3:** Study, game, and assessment timing characteristics of the studies.

Characteristics	Present		Absent	
	*N* Obs	*N* Studies	*N* Obs	*N* Studies
Study				
Games Included	1,843	6	3,963	16
Quota Sampling	4,353	11	1,453	11
English Language	2,049	4	3,757	18
Task				
Deception	249	2	1,594	4
Incentivization	743	3	1,100	3
Assessment Timing				
Two-week time interval	811	4	1,032	2

Note. “Present” and “Absent” indicate whether the respective characteristic was implemented in a given study. “N Obs” refers to the number of participants (observations), and “N Studies” to the number of independent studies. A more detailed table with information on study level is provided in the online supplement.

### Material

The **Facets of Altruistic Behaviors (FAB) scale**^48^ and its English translation (see Supplement S1) were used to assess the three behavioral traits of Help Giving (HG), Peer Punishment (PP), and Moral Courage (MC). The FAB scale was constructed using an ant colony optimization approach^155^, enabling the creation of a highly reliable short scale. Each subscale consists of five items that vary in their focus; together, they aim to assess one of the three traits as self-reported behaviors. HG describes helping and sharing without expecting anything in return. Costs involved range from small donations or favors to risking one’s well-being or life. Example items are: “I often do good things for others without reservation or expectations” and “In an emergency I would probably spontaneously risk my own life to save complete strangers.” Items on the PP subscale are closely related to Public Goods Game scenarios in which a free rider exploits the community’s cooperation for their own benefit. Punishing this behavior, even if it is costly for the individual, helps to defend and safeguard societal norms, benefiting all group members. However, costs are calculable, as punishment is mostly anonymous or coordinated with others. Example of PP items include “When someone purposely takes advantage of the community, I discretely get back at them in one way or another.” and “When someone demands special privileges for themselves, I look for others with whom I can try to stop them.” MC describes standing up for one’s moral values when faced with imminent social threats. Unlike PP, moral courageous behavior is proactive and visible. It advocates for innovation and transformation of societal norms to align with one’s moral ideals, even when facing pressure and threats from the majority or powerful individuals. Examples of MC items include “I openly question the decisions of authorities or superiors.” and “It has happened that I have affronted others due to my moral convictions.” All items on the FAB scale refer to actual behaviors, in order to minimize social desirability bias and maximize predictive validity. However, since some of these behaviors are rare yet highly indicative, some items are written in the subjunctive (i.e., “I would jeopardize my own well-being in order to help the sick and hungry.”). Items are answered using a six-point Likert scale, where 1= *strongly disagree*, 2 = *disagree*, 3 = *rather disagree*, 4 = *rather agree*, 5 = *agree*, and 6 = *strongly agree*. The subscales showed composite reliabilities of ω = 0.74–0.79 (Windmann et al^48^. report ω = 0.75–0.76).

The **Dictator Game** (DG) was used to assess altruistic sharing in six studies. In each study, the participants were randomly selected as the winner of this decision situation. They received a gift of $10.70 (for participants in the US) or €10.70 (for participants in Germany). They could keep the money or share the amount with another unknown participant who had not been randomly selected as winner. Similar to HG, there was no expectation of receiving anything in return because the DG was one-shot and anonymous. We assessed the willingness to share and the amount shared.

The **Public Goods Game** (PGG) **with punishment option** was used to assess costly punishment in six studies. To improve comprehension, we added pictorial descriptions of the game process, including examples, across studies. Participants were part of a group of four pursuing an investment project. They were given an initial endowment of $5 (€5) and asked to invest in the project. The sum of all contributions was then tripled and distributed equally among all members. Participants could punish one group member who contributed less than their fair share but still benefited from the group’s investments. Punishing a group member reduced the outcome by a fee-to-fine ratio of 1:3. Similar to PP, participants are part of a group with cooperation norms that are violated. Punishment remains anonymous, making the costs calculable. We assessed the likelihood to punish, as well as the costs invested in punishment.

The **Ultimatum Game** (UG) was also used to assess peer punishment in four studies. In all four studies, participants were first asked to accept or reject an unfair offer of $2.90 (€2.90) from another person (on a peer level) who had been given $9.50 (€9.50). If they accepted, they would receive $2.90 (€2.90) and the other person would keep $6.60 (€6.60). If they rejected, both would receive nothing. In three studies, they were then asked to indicate their Minimal Acceptable Offer (MAO) between $0 (€0) and $9.50 (€9.50) which indicates the threshold above which participants would accept any offer. Unlike PP, the UG does not include a group setting. However, it has simpler rules and is therefore less susceptible to comprehension bias compared to PGG. We assessed the likelihood to reject the unfair offer and the MAO.

The **Third-Party Intervention Paradigm **(introduced within the same research program by Windmann et al.^48^) was used in six studies to assess morally courageous behavior in a controlled situation, aligned with the conceptualization of moral courageous behavior in the FAB scale. Participants observed another participant who was powerful by chance and had the right to decide about their own and others’ payouts. This powerful participant kept a large sum of $9.40 (€9.40), that could have been shared with others, then took $11.50 (€11.50) from other participants without justification, and excluded others from payout. The participants, as well as three other participants, were given the option of sending a message to this unfair person, at the risk of being excluded from the game and the payout. The three other participants did not send a message to the offender, suggesting that the other participants accepted the person’s unfair behavior. We then asked participants if they wanted to send the offender a message, one that would be displayed to all participants in this game. If yes, a text field for the message followed. Similar to MC, intervening against a powerful and legitimate person in order to change the normative system according to one’s own moral standards is visible and associated with personal risk. We assessed the likelihood to send a message to the offender.

### Procedure

All studies were administered online and took place from 2019 to 2025. The studies that involved only the self-report FAB scale but not the economic games had various research goals that are not detailed here. However, more information can be found in their preregistrations in the online supplement. Items from the FAB scale were always presented in a random order, sometimes mixed with items from other scales and attention check questions. Informed consent was appropriately obtained from all participants.

Across those studies that included the games, some characteristics were varied (see Table 3). In half of the studies, participants were deceived to believe that the other players, with whom they interacted, were real other participants. In these studies, participants were disclosed after participation. In the other studies, they were disclosed beforehand and were asked to act as if there were other real participants involved. However, the games were designed to feel as realistic as possible. For example, brief pauses simulated other players contemplating. In three studies, participants were incentivized based on their game behavior. In two studies, there was payment for participation but it was independent of their decisions, one study did not include any payment. In five studies, participants first responded to the FAB scale and then to the games. In three of these studies, there were around 10–14 days between answering the FAB scale and the games, in the two other studies, there was no time interval between the two study parts. In one study, the presentation of games and FAB scale was randomized, with 10–14 days between the two parts.

### Statistical analysis

#### Preprocessing

For all analyses presented in this article, we used R, Version 4.5.0^156^, in R-Studio, Version 2025.5.0.496^157^. Raw data of all 22 studies were preprocessed as follows. Variables were renamed and recoded to make data comparable across studies. Then, we excluded participants in line with the preregistered criteria. For those three studies that were not preregistered, we used the same criteria that were used in prior and similar studies. R scripts with data exclusion and preprocessing can be found in the online supplement for each study.

#### Model fit evaluation and factor score extraction

We used a Confirmatory Factor Analysis (CFA) to check the model fit of the three-factorial FAB scale. Following recommendations of Hu and Bentler^158^, we used the cutoff values of SRMR ≤ 0.08, RMSEA ≤ 0.06, and CFI ≥ 0.95 to evaluate the quality of the defined model. We then derived the regression factor scores for the FAB subscales (based on 5,806 participants), as implemented in *lavaan*^159^, in order to enhance the precision of estimates when predicting the observed game behaviors in the next step.

#### Prediction of game behaviors

We analyzed the game behaviors separately and accounted for the nesting of participants in studies by using mixed effects model where applicable. Because Dictator Game sharing and punishment costs indicated a high positive skew with predominating zeroes (35.2% of Dictator Game sharing and 59.6% of punishment costs), we used a two-part (hurdle) mixed-model. In a first step, we computed a generalized linear mixed-effects model with logit link that assessed the likelihood of sharing and punishing, followed by a linear mixed-effects model for the conditional distribution of positive values. We applied log transformation to the positive values of sharing amount and punishment costs to improve the approximation to normality of the residuals. To aid interpretation, we exponentiated the coefficients and expressed them as a percentage change on the original scale. For third-party intervening, we fitted a generalized linear mixed-effects model with logit link to account for the binary data.

We first computed the intercept-only model to determine the intraclass correlation coefficients (ICC). Then, we included the factor scores of the subscales HG, PP, and MC on person level (level 1) and the binary predictor deception on group level (level 2), as well as its interaction with the factor score that was aimed at predicting the game behavior. Unfortunately, we could not include the predictors of incentivization and two-week time interval in our models to investigate interaction effects with the factor score on game behavior because they were varied almost parallel to each other and were therefore highly collinear.

As only three studies included data on the likelihood to reject and four studies included the MAO in the Ultimatum Game, estimating mixed-effects models was not suitable. Instead, we calculated a generalized linear model (binomial family, logit link) for the likelihood to reject and a linear model with robust standard errors to account for non-normal residuals and heteroscedasticity for the MAO. We included the regression factor scores of the HG, PP, and MC subscales, the predictor deception, and the PP × deception interaction in the models.

#### Correlations within and across methods

To facilitate comparison with prior studies, we additionally computed pairwise Spearman correlations with Holm-adjusted *p*-values between self-report measures and game behaviors. Specifically, we examined correlations with the amount shared in the Dictator Game (including zeros), punishment costs in the Public Goods Game (including zeros), rejection likelihood and MAO in the Ultimatum Game, and intervention likelihood in the Third-Party Intervention Paradigm. Notably, these correlations should be interpreted with caution: due to the substantial number of zero values in the distributions of Dictator Game sharing and punishment costs, the resulting Spearman coefficients are expected to be inflated relative to the more realistic effect estimates obtained from the model-based analyses reported above. As a general guideline, correlations of approximately *r*=.20 can be considered medium and practically meaningful, whereas correlations around *r*=.30 represent comparatively large effects^120^.

## Data Availability

All datasets and other supplementary files are available on OSF at https://doi.org/10.17605/OSF.IO/T5MF .
